# Case Report: A novel likely pathogenic *GCK* variant in a young Chinese girl with severe insulin resistance

**DOI:** 10.3389/fendo.2026.1826357

**Published:** 2026-04-29

**Authors:** Haoyu Bai, Jie Yu, Tong Wang, Jing Ren, Chuhan Shao, Qian Zhang, Xinhua Xiao

**Affiliations:** 1 NHC Key Laboratory of Endocrinology (Peking Union Medical College Hospital), Diabetes Research Center of Chinese Academy of Medical Sciences, Department of Endocrinology, Peking Union Medical College Hospital, Chinese Academy of Medical Sciences & Peking Union Medical College, Beijing, China; 2 Department of Endocrinology, The 305 Hospital of PLA, Beijing, China

**Keywords:** GCK-MODY, glucokinase, glutamic acid decarboxylase antibody, novel variant, type 2 diabetes, whole-exome sequencing

## Abstract

**Objective:**

To report a novel *GCK* variant (c.263T>C) identified in a patient with glucokinase-maturity-onset diabetes of the young (GCK-MODY) and to evaluate its pathogenicity and potential structural impact using *in silico* analyses. The proband had a paternal history of type 2 diabetes mellitus (T2DM) and was overweight with marked insulin resistance, presenting with clinical features suggestive of T2DM. She also exhibited transient glutamic acid decarboxylase antibody (GADA) positivity. Clinical characteristics were further evaluated to facilitate the differential diagnosis.

**Case presentation:**

A novel *GCK* missense variant was identified in a Chinese family, with the daughter as the proband. At 6 years of age, she presented with mild fasting hyperglycemia and initial GADA positivity and was misdiagnosed with type 1 diabetes mellitus (T1DM). However, her clinical features and family history were suggestive of MODY. Whole-exome sequencing revealed a heterozygous *GCK* c.263T>C variant, confirmed by Sanger sequencing in her mother, maternal grandmother, and maternal grandaunt. The variant was classified as likely pathogenic, establishing a diagnosis of GCK-MODY. Notably, she also exhibited obesity and a paternal family history of T2DM. During follow-up, GADA seroconverted to negative, with no evidence of persistent autoimmune diabetes.

**Conclusion:**

This case identifies a novel likely pathogenic *GCK* variant (c.263T>C) in a Chinese family and highlights the coexistence of GCK-MODY with insulin resistance within the same pedigree. The proband’s severe insulin resistance may be attributable to the combined effects of obesity and a paternal history of T2DM, contributing to a complex clinical phenotype. Transient GADA positivity was not associated with persistent autoimmune diabetes, suggesting it may reflect metabolic stress rather than true autoimmune pathogenesis.

## Introduction

Maturity-onset diabetes of the young (MODY) is a monogenic form of diabetes characterized by substantial genetic heterogeneity and an estimated prevalence of approximately 1 in 10,000 in adults and 1 in 23,000 in children (1). It typically presents before the age of 25, follows an autosomal dominant inheritance pattern, and is primarily caused by impaired pancreatic β-cell function (2). Due to overlapping clinical features, MODY is frequently misdiagnosed as type 1 diabetes mellitus (T1DM) or type 2 diabetes mellitus (T2DM) (3, 4).

To date, at least 14 MODY subtypes have been identified, each caused by mutations in distinct genes (5). Glucokinase (*GCK*), the first gene associated with monogenic diabetes (6), underlies one of the most common MODY subtypes, termed GCK-MODY (7, 8). *GCK* encodes a key glucose sensor in pancreatic β cells that regulates insulin secretion in response to blood glucose levels. More than 1,200 *GCK* variants, including missense, nonsense, frameshift, splice-site, and untranslated region mutations, have been reported in ClinVar. Although GCK-MODY is classically associated with mild, stable fasting hyperglycemia, its clinical presentation may be heterogeneous, particularly in the presence of additional metabolic factors.

MODY has traditionally been regarded as a non-autoimmune form of diabetes; however, diabetes-associated autoantibodies have occasionally been detected (9). The clinical relevance of autoantibody positivity in MODY remains unclear, especially when accompanied by features of insulin resistance (IR) or a family history of T2DM, which may further complicate differential diagnosis.

Here, we report a Chinese girl harboring a novel *GCK* missense variant (c.263T>C, p.Leu88Pro) who presented with asymptomatic mild fasting hyperglycemia and a three-generation family history of diabetes. *In silico* analyses supported the likely pathogenicity of the variant, consistent with a diagnosis of GCK-MODY. Notably, features suggestive of IR, together with a paternal family history of T2DM, indicated phenotypic overlap with T2DM. Transient glutamic acid decarboxylase antibody (GADA) positivity was also observed; however, preserved β-cell function and subsequent seronegativity were not consistent with autoimmune diabetes.

## Case presentation

In February 2023, a 6-year-old girl was found to have elevated fasting blood glucose during postoperative evaluation following papillary thyroid carcinoma resection at an outside hospital. Her fasting blood glucose (FBG) was 6.15 mmol/L. Her height was 130 cm and her weight was 23 kg (+1 SD), with a body mass index (BMI) of 13.61 kg/m². She was diagnosed with impaired fasting glucose. She denied polydipsia, polyphagia, polyuria, or weight loss. No treatment was initiated, and FBG remained stable between 6.3 and 6.5 mmol/L during follow-up.

In January 2024, repeat evaluation showed an FBG of 7.51 mmol/L and glycated hemoglobin (HbA1c) of 6.4%. Fasting C-peptide was 2.36 ng/mL (reference range: 0.80–4.20 ng/mL), and fasting insulin was 17.9 μIU/mL (reference range: 3.0–25.0 μIU/mL). The homeostasis model assessment of insulin resistance (HOMA-IR) was 5.97. Islet autoantibody testing showed insulin autoantibodies (IAA) of 0.71 COI, islet cell antibodies (ICA) of 0.57 COI, GADA of 15.83 IU/mL, and tyrosine phosphatase-like protein islet antigen 2 antibodies (IA-2A) of 9.27 IU/mL.

At that time, her height was 131.3 cm, and her weight was 33.5 kg (+3SD), corresponding to a BMI of 19.43 kg/m², which exceeded the age- and sex-specific threshold for obesity (18.9 kg/m^2^) (10). Her waist circumference was 70 cm and her hip circumference was 74 cm, yielding a waist-to-hip ratio of 0.95. She appeared overweight, without Cushingoid features, dysmorphic characteristics, or acanthosis nigricans.

**Figure 1:**
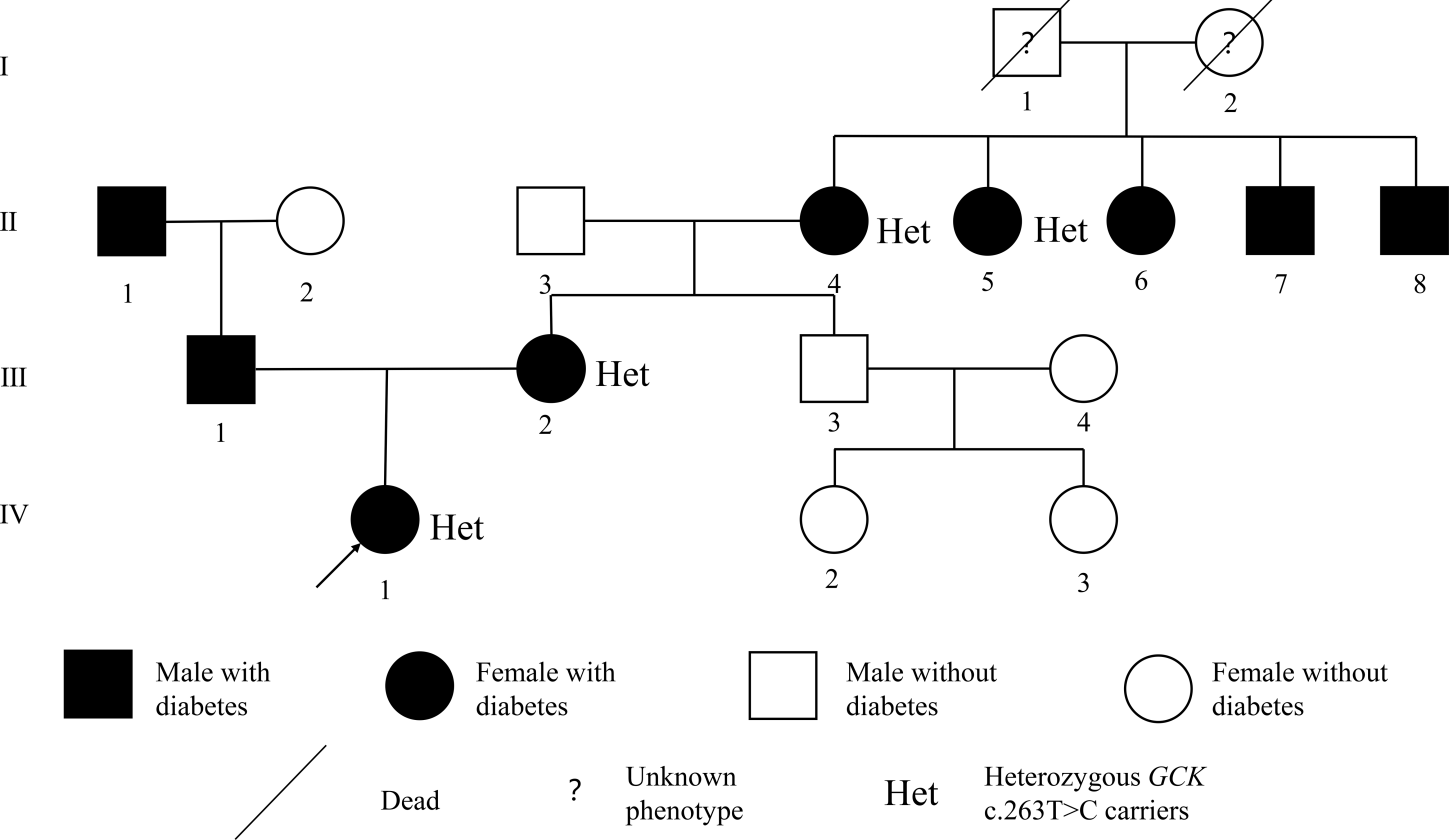
Pedigree of the family with the *GCK* variant. The proband is indicated by the arrow. Squares represent male subjects and circles represent female subjects. Black-filled symbols indicate individuals diagnosed with diabetes, whereas unfilled symbols indicate individuals without diabetes. Diagonal lines denote deceased individuals, and question marks indicate unknown diabetes status. “Het” denotes individuals heterozygous for the GCK variant (c.263T>C). The proband’s father was diagnosed with type 2 diabetes during follow-up.

She was born at term by cesarean section with a birth weight of 3.5 kg. Her mother was diagnosed with gestational diabetes mellitus (GDM) at 20 weeks of gestation (at 28 years) and did not receive pharmacological treatment. Both her mother and maternal grandmother had mild fasting hyperglycemia without medication. Her paternal grandfather had T2DM and was treated with metformin, and her father was diagnosed with T2DM at 39 years of age. The clinical characteristics of her parents are summarized in **Table 1**, and the pedigree is shown in **Figure 1**.

**Table 1:** Clinical characteristics and laboratory findings of the proband’s parents.

Clinical features	Reference range	Mother	Father
Age at diagnosis (years)	–	28	39
BMI at diagnosis (kg/m^2^)	18.5–23.9	22.49	27.78
Hypertension	–	No	Yes
FBG (mmol/L)	3.9–6.1	7.13	7.47
HbA1c (%)	4.5–6.3	6.4	6.1
Triglycerides (mmol/L)	0.45–1.70	1.32	3.06
Total cholesterol (mmol/L)	1.80–5.17	4.24	5.96
LDL-C (mmol/L)	<3.15	2.70	4.05
hs-CRP (mg/L)	<3.00	<0.20	NA
Treatment	–	Diet	Diet
Onset	–	Gestational diabetes mellitus	Hyperglycemia detected during routine medical examination

BMI, body mass index; FBG, fasting blood glucose; HbA1c, glycated hemoglobin; hs-CRP, high-sensitivity C-reactive protein; LDL-C, low-density lipoprotein cholesterol; NA, not available.

She subsequently presented to our outpatient clinic for further evaluation. Repeat testing for IAA, ICA, GADA, and IA-2A was negative.

Given the early onset of hyperglycemia and the family history across three generations, genetic testing was performed. After obtaining written informed consent from both parents, peripheral blood samples were collected from the patient and her parents. Genomic DNA was extracted, and whole-exome sequencing (WES) was performed in the proband, followed by Sanger sequencing in her parents. The reference genome was GRCh38/hg38.

**Figure 2:**
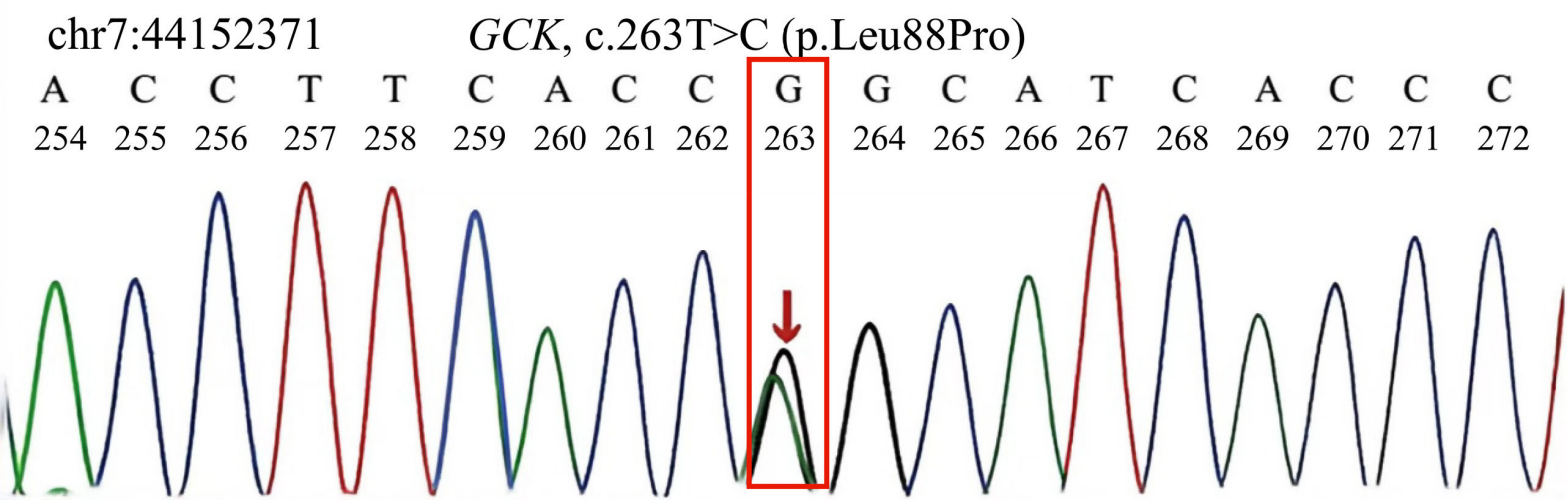
Identification and validation of the *GCK* variant in the proband. Whole-exome sequencing identified a heterozygous variant (c.263T>C) in the *GCK* gene, which was subsequently confirmed by Sanger sequencing. The chromatogram shows overlapping peaks at the variant site, consistent with heterozygosity. The red arrow indicates the position of the mutated nucleotide. This variant results in a missense change (p.Leu88Pro) based on the reference transcript NM_000162.5.

A heterozygous c.263T>C variant in the *GCK* gene (NM_000162.5) was identified, resulting in a leucine-to-proline substitution at codon 88 (p.Leu88Pro). The variant was detected in both the proband and her mother. Sanger sequencing of additional maternal relatives showed that the maternal grandmother and maternal great-aunt also carried the same variant. The WES findings are shown in **Figure 2**.

According to the American College of Medical Genetics and Genomics and the Association for Molecular Pathology (ACMG/AMP) guidelines (11), the variant was classified as likely pathogenic. Based on the genetic findings and clinical presentation, the patient was diagnosed with GCK-MODY. The patient was managed with dietary modification and regular exercise and continues to undergo regular follow-up. The laboratory findings are summarized in **Table 2**.

**Table 2:** Longitudinal laboratory findings of the proband during follow-up.

Clinical features	Reference range	March 2024	July 2024	January 2025	August 2025	January 2026
Age (years)	–	7	7	8	8	9
BMI (kg/m^2^)	–	19.43	19.49	19.49	19.11	17.83
FBG (mmol/L)	3.9–6.1	6.3	7.4	6.7	7.1	6.8
PBG, 2 h (mmol/L)	<7.8	7.2	8.2	NA	NA	NA
HbA1c (%)	4.5–6.3	6.6	6.7	6.3	6.3	6.3
Fasting insulin (μIU/mL)	1.8–25.0	NA	NA	NA	14.5	7.3
Fasting C-peptide (ng/mL)	0.80–4.20	1.01	5.56	1.80	1.96	1.13
C-peptide, 2 h (ng/mL)	–	3.67	2.40	NA	NA	NA
hs-CRP (mg/L)	<3.00	5.02	0.70	0.99	NA	0.77
Triglycerides (mmol/L)	0.45–1.70	0.89	1.00	1.56	1.24	0.51
LDL-C (mmol/L)	<2.59	2.55	2.36	2.70	2.58	2.72
Creatinine (μmol/L)	18–69	29	27	31	NA	34
TPOAb (IU/mL)	<60	28	72	14.8	NA	NA
TgAb (IU/mL)	<4.5	1.3	1.3	1.3	1.3	1.3

Fasting glucose, insulin, and C-peptide levels refer to serum measurements obtained in the fasting state. PBG, 2 h and C-peptide, 2 h indicate measurements obtained 2 h after glucose load. BMI cutoff values for pediatric overweight were based on the Chinese national standard (GB/T 31178–2014): 7–8 years, ≥17.2 kg/m²; 8–9 years, ≥18.1 kg/m²; 9–10 years, ≥18.9 kg/m².

BMI, body mass index; FBG, fasting blood glucose; PBG, postprandial blood glucose; HbA1c, glycated hemoglobin; hs-CRP, high-sensitivity C-reactive protein; LDL-C, low-density lipoprotein cholesterol; TPOAb, thyroid peroxidase antibody; TgAb, thyroglobulin antibody; NA, not available.

## Discussion

In this study, we report a young girl with clinical features suggestive of GCK-MODY, in whom WES identified a novel likely pathogenic *GCK* variant (c.263T>C, p.Leu88Pro). GCK-MODY is typically characterized by mild, stable fasting hyperglycemia with preserved β-cell function; however, the proband also exhibited obesity and marked IR, which are atypical for this condition. In addition, transient positivity of GADA was observed, further complicating the clinical presentation.

The clinical diagnosis of MODY is typically based on early-onset diabetes (usually before 25 years of age), autosomal dominant inheritance, absence of obesity, and clinical features not consistent with T1DM or T2DM (12). Patients with GCK-MODY generally exhibit persistent mild fasting hyperglycemia (5.4–8.3 mmol/L), HbA1c levels of 5.8%–7.6%, preserved β-cell function, and a low risk of microvascular complications (13). GCK-MODY represents the most common subtype of monogenic diabetes in pediatric populations (14). Accordingly, genetic testing is recommended in children with stable, incidental hyperglycemia without progressive deterioration (15).

The *GCK* gene, located on chromosome 7p13, encodes glucokinase, a key glucose sensor in pancreatic β cells and hepatocytes that regulates insulin secretion and hepatic glycogen synthesis (6). Pathogenic variants typically reduce enzymatic activity through impaired catalytic function, decreased protein stability, or altered interaction with glucokinase regulatory protein (GCKR), thereby shifting the glucose threshold for insulin secretion upward (approximately 6.5–7.5 mmol/L) and resulting in mild chronic hyperglycemia (16). Heterozygous loss-of-function variants are the molecular basis of GCK-MODY (17).

In this study, we identified a previously unreported heterozygous variant, c.263T>C(p.Leu88Pro), classified as likely pathogenic according to the 2015 ACMG/AMP guidelines. This classification is supported by multiple lines of evidence. The variant is absent from major population databases (1000 Genomes, gnomAD, ExAC) and ClinVar/VarCards, indicating its rarity. Computational predictions consistently indicated a deleterious effect, including CADD (18), REVEL (19), AlphaMissense (20), PolyPhen-2, SIFT, PROVEAN, and MutationTaster (**Supplementary Table 1**). In addition, the variant segregates with hyperglycemia across three generations, consistent with autosomal dominant inheritance. To further explore its potential structural impact, *in silico* modeling was performed. Although secondary and homology modeling suggested local conformational changes near residues 88 and 157 (21, 22), these predicted alterations do not establish a definitive functional consequence, reflecting the limitations of computational approaches. Nevertheless, the combined evidence from rarity, *in silico* prediction, and co-segregation strongly supports the likely pathogenicity of this variant.

The clinical spectrum of T2DM-related metabolic features in individuals with GCK-MODY remains incompletely defined, with some patients exhibiting central adiposity, hypertension, or dyslipidemia, thereby complicating phenotypic classification (23, 24). In the present case, the proband exhibited persistent mild fasting hyperglycemia from the age of 6 years, along with obesity and marked IR, but did not meet the diagnostic criteria for metabolic syndrome. A three-generation family history further supported a clinical suspicion of GCK-MODY. Notably, the maternal lineage demonstrated classical GCK-MODY features, including early-onset mild hyperglycemia, normal BMI, and stable glycemia without pharmacological intervention. In contrast, the paternal lineage showed typical manifestations of T2DM, characterized by overweight status, metabolic abnormalities, and the need for oral hypoglycemic agents. These divergent familial patterns likely contributed to the complex metabolic phenotype observed in the proband.

The coexistence of childhood obesity likely plays a central role in the development of IR, as excess visceral adiposity promotes lipolysis, increased free fatty acid release, and pro-inflammatory adipokine secretion, all of which impair insulin signaling (25). In parallel, the paternal family history of T2DM suggests an inherited predisposition to IR. Consistent with this, Ji et al. reported that individuals with GCK-MODY may develop more severe diabetic phenotypes when accompanied by a high polygenic risk score for IR and T2DM, highlighting the additive effect of polygenic background on monogenic diabetes manifestations (26). Taken together, the IR observed in this proband is likely the result of a combined effect of obesity-related metabolic dysregulation and inherited genetic susceptibility. This case underscores the metabolic heterogeneity of GCK-MODY and suggests that additional genetic and environmental factors may significantly modify its clinical phenotype.

Although MODY is traditionally considered a non-autoimmune form of diabetes, islet autoantibodies have occasionally been detected in patients with MODY (27–31), with a prevalence comparable to that observed in healthy controls (32). McDonald et al. reported that GADA positivity may occur in MODY, whereas IA-2A is rarely detected, supporting its utility in excluding MODY (32). Their analysis primarily included MODY caused by mutations in *GCK*, hepatocyte nuclear factor 1 alpha (*HNF1A*), and hepatocyte nuclear factor 4 alpha (*HNF4A*). In contrast, other studies have identified IAA and ICA in a subset of patients, some of whom exhibited clinical features consistent with T1DM, raising the possibility of coexistence in selected cases (33).

Several studies have suggested that metabolic stress may contribute to transient islet autoantibody positivity (9). Chronic hyperglycemia and increased insulin demand can place stress on β cells, potentially leading to cellular dysfunction or apoptosis and the release of intracellular antigens. These antigens may subsequently be presented to the immune system, transiently activating autoreactive B cells and resulting in temporary GADA production. Such responses are typically mild and self-limiting, which is consistent with observations that autoantibody titers often decline with improved glycemic control.

In the present case, the proband initially exhibited GADA positivity, which led to a misdiagnosis of T1DM. However, the absence of progressive β-cell dysfunction and subsequent conversion to autoantibody negativity were not consistent with persistent autoimmune diabetes. Instead, the transient GADA positivity is more likely to reflect β-cell stress induced by hyperglycemia, with later seronegativity corresponding to metabolic stabilization rather than resolution of an underlying autoimmune process. Additionally, the proband exhibited a mildly elevated hs-CRP level (5.02 mg/L in March 2024), which appeared to be transient and likely reflected subclinical inflammation. Such a mild and transient elevation is unlikely to be clinically meaningful or to influence the characteristic metabolic phenotype of GCK-MODY.

For patients with GCK-MODY and IR, lifestyle modifications remain the mainstay of management during non-pregnant periods, as pharmacological interventions such as oral hypoglycemic agents or insulin generally provide limited additional glycemic benefit (34, 35). Dietary intervention alone can achieve modest yet sustained reductions in glucose levels without escalation of medical therapy (36). In the present case, given the preserved β-cell function and stable mild fasting hyperglycemia, conservative management was adopted. During follow-up, dietary and exercise interventions stabilized fasting glucose and HbA1c, accompanied by a reduction in BMI from 19.11 to 17.83 kg/m² and an improvement in IR (HOMA-IR 4.57 to 2.21).

This study provides three main insights. First, we report a novel *GCK* variant in a Chinese pedigree, supported by computational and structural analyses as likely pathogenic, thereby expanding the mutational spectrum of GCK-MODY. Second, this case illustrates that GCK-MODY may present with additional metabolic features associated with IR, particularly in the context of obesity and a paternal T2DM background, which may complicate phenotypic classification. Third, the transient GADA positivity observed in this patient suggests that islet autoantibodies in MODY do not necessarily indicate autoimmune diabetes, underscoring the need for cautious interpretation of serological findings in atypical pediatric diabetes.

However, this study has several limitations. Most notably, no functional validation of the identified variant was performed, either *in vitro* or *in vivo*, and further studies are required to confirm its pathogenic role.

## Conclusion

In conclusion, we report a novel likely pathogenic *GCK* variant (c.263T>C) identified in a Chinese pedigree. The maternal lineage exhibited clinical features consistent with classical GCK-MODY, whereas the paternal lineage was characterized by T2DM. The proband presented with severe IR, which may be attributed to the combined influence of obesity and a paternal T2DM background, contributing to phenotypic heterogeneity. In addition, transient GADA positivity was observed without evidence of persistent autoimmune diabetes, suggesting that this finding may have been related to metabolic stress rather than an underlying autoimmune mechanism.

## Data Availability

The original contributions presented in the study are included in the article/**Supplementary Material**. Further inquiries can be directed to the corresponding author.
