# Supplementary material for: Case Report: A novel likely pathogenic GCK variant in a young Chinese girl with severe insulin resistance
Source: Front Endocrinol (Lausanne). 2026 Apr 29;17:1826357. doi: 10.3389/fendo.2026.1826357 (PMC13167591; doi:10.3389/fendo.2026.1826357)
Supplement: Supplementary file 1 [file SupplementaryFile1.docx]

Supplementary Material

# Supplementary Figures and Tables

## Supplementary Figures


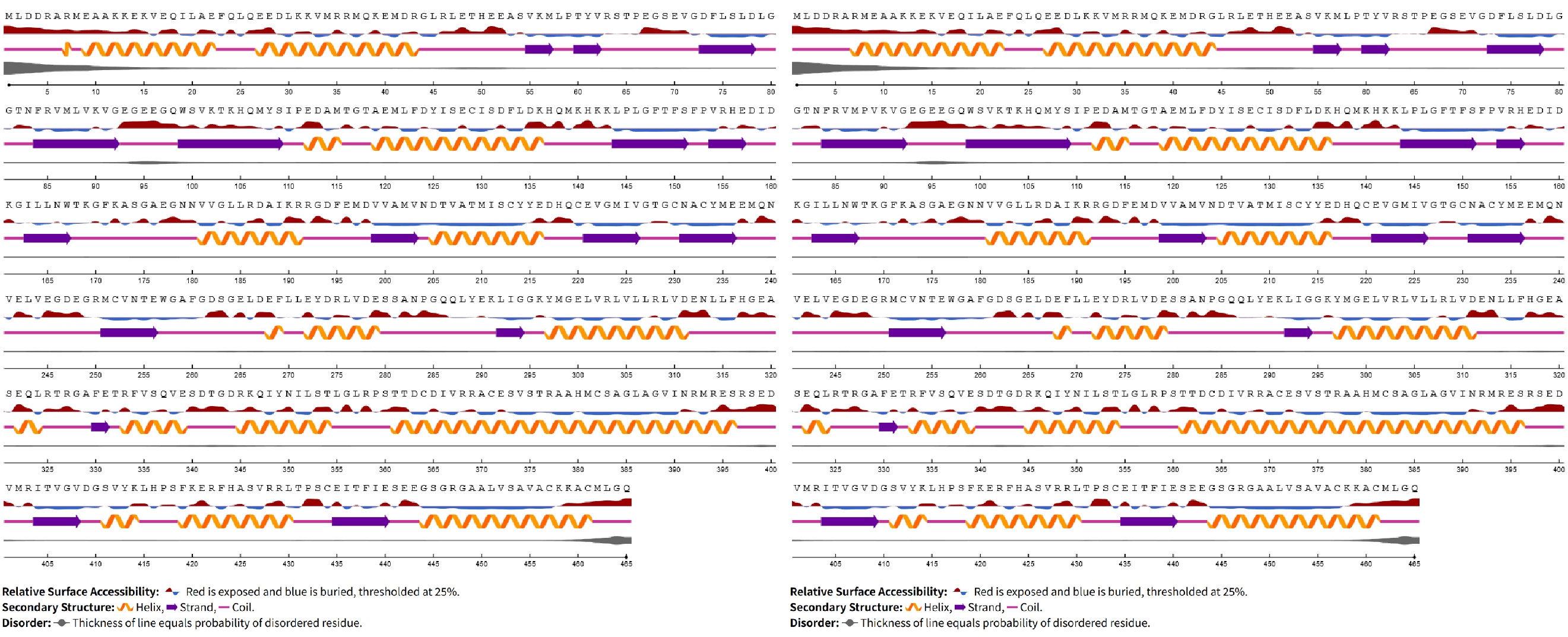


**Supplementary Figure 1.** Secondary structure prediction of wide-type glucokinase (left) and the L88P variant (right) by NetSurfP-3.0. No change was observed at residue 88, while an extension of a β-strand at residue 157 was suggested in the variant.

Abbreviations: L, Leu (leucine); P, Pro (proline).


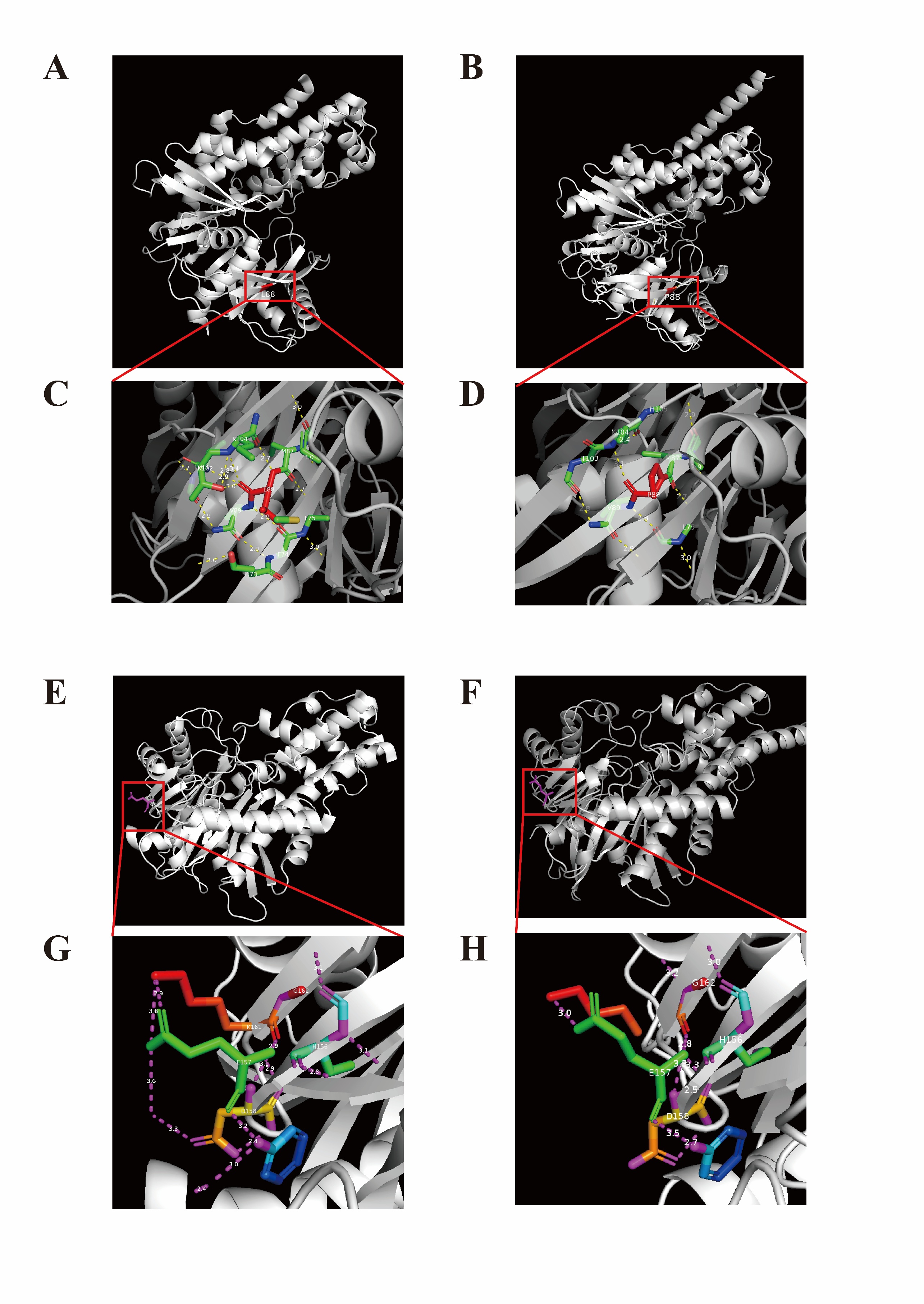


**Supplementary Figure 2.** Predicted three-dimensional structures of wild-type glucokinase and the L88P variant generated by Swissmodel (GMQE 0.92, QMEANDisCo 0.87 ± 0.05) and visualized using Pymol (version 3.1.0). Panels A and E show the overview structure of wild-type glucokinase, while panels B and F show the overall structure of the L88P variant. Panels C and D illustrate the local structure around residue 88 in the wild-type and variant, respectively, and panels G and H show the corresponding regions around residue 157. These analyses indicate local conformational differences and altered residue interactions in regions surrounding residues 88 and 157.

Abbreviations: L, Leu (leucine); P, Pro (proline); K, Lys (lysine); T, Thr (threonine); M, Met (methionine); D, Asp (aspartic acid); V, Val (valine); H, His (histidine); F, Phe (phenylalanine).

## Supplementary Tables

**Supplementary Table 1.** The prediction results regarding the deleterious impact of the variant on protein function

|  | PolyPhen-2 | SIFT | PROVEAN | MutationTaster |
| --- | --- | --- | --- | --- |
| Results | Probably damaging | Deleterious | Deleterious | Deleterious |

Abbreviations: PolyPhen-2 (Polymorphism Phenotyping v2), a tool for predicting the potential impact of amino acid substitutions on protein structure and function based on physical and comparative considerations; SIFT (sorting intolerant from tolerant), a tool that predicts whether an amino acid substitution affects protein function based on sequence homology and physical properties; PROVEAN (Protein Variation Effect Analyzer), a tool used to assess the [potential functional impact of amino acid substitutions or indels on proteins.

# Web Resourses

ClinVar, <https://www.ncbi.nlm.nih.gov/clinvar/>

1000 Genomes, <http://browser.1000genomes.org>/

gnomAD, <https://gnomad.broadinstitute.org/>

ExAC, <http://exac.broadinstitute.org/>

VarCards, <https://genemed.tech/varcards2/#/index/home>

CADD v1.7, <https://cadd.gs.washington.edu/snv>

Revel, <https://sites.google.com/site/revelgenomics/>

PolyPhen-2, <http://genetics.bwh.harvard.edu/pph2/>

SIFT, <https://sift.bii.a-star.edu.sg/>

PROVEAN, <http://provean.jcvi.org/>

MutationTaster, <https://www.genecascade.org/MutationTaster2021/#transcript>

AlphaMissense, <https://alphamissense.hegelab.org/>

UniProt, <https://www.uniprot.org/>

NetSurfP-3.0, <https://services.healthtech.dtu.dk/services/NetSurfP-3.0/>

Swissmodel, <https://swissmodel.expasy.org/>
